# Twitter discussions on breastfeeding during the COVID-19 pandemic

**DOI:** 10.1186/s13006-023-00593-x

**Published:** 2023-11-04

**Authors:** Jawahar Jagarapu, Marlon I. Diaz, Christoph U. Lehmann, Richard J. Medford

**Affiliations:** 1 Department of Pediatrics, University of Texas Southwestern Medical Center, Dallas, TX USA; 2 School of Biomedical Informatics, University of Texas, Houston, TX USA; 3 Division of Neonatal-Perinatal Medicine, UT Southwestern Medical Center, 5323 Harry Hines Blvd, Suite F3.118, Dallas, TX 75390 USA; 4 Center for Clinical Informatics, University of Texas Southwestern Medical Center, Dallas, TX USA; 5 Paul L. Foster School of Medicine, Texas Tech University Health Sciences Center, El Paso, TX USA; 6 Department of Internal Medicine, University of Texas Southwestern Medical Center, Dallas, TX USA

**Keywords:** Social media, Twitter, Breastfeeding, COVID-19, Breast milk, Lactation, Sentiment analysis

## Abstract

**Background:**

Breastfeeding is a critical health intervention in infants. Recent literature reported that the COVID-19 pandemic resulted in significant mental health issues in pregnant and breastfeeding women due to social isolation and lack of direct professional support. These maternal mental health issues affected infant nutrition and decreased breastfeeding rates during COVID-19. Twitter, a popular social media platform, can provide insight into public perceptions and sentiment about various health-related topics. With evidence of significant mental health issues among women during the COVID-19 pandemic, the perception of infant nutrition, specifically breastfeeding, remains unknown.

**Methods:**

We aimed to understand public perceptions and sentiment regarding breastfeeding during the COVID-19 pandemic through Twitter analysis using natural language processing techniques. We collected and analyzed tweets related to breastfeeding and COVID-19 during the pandemic from January 2020 to May 2022. We used Python software (v3.9.0) for all data processing and analyses. We performed sentiment and emotion analysis of the tweets using natural language processing libraries and topic modeling using an unsupervised machine-learning algorithm.

**Results:**

We analyzed 40,628 tweets related to breastfeeding and COVID-19 generated by 28,216 users. Emotion analysis revealed predominantly “Positive emotions” regarding breastfeeding, comprising 72% of tweets. The overall tweet sentiment was positive, with a mean weekly sentiment of 0.25 throughout, and was affected by external events. Topic modeling revealed six significant themes related to breastfeeding and COVID-19. Passive immunity through breastfeeding after maternal vaccination had the highest mean positive sentiment score of 0.32.

**Conclusions:**

Our study provides insight into public perceptions and sentiment regarding breastfeeding during the COVID-19 pandemic. Contrary to other topics we explored in the context of COVID (e.g., ivermectin, disinformation), we found that breastfeeding had an overall positive sentiment during the pandemic despite the documented rise in mental health challenges in pregnant and breastfeeding mothers. The wide range of topics on Twitter related to breastfeeding provides an opportunity for active engagement by the medical community and timely dissemination of advice, support, and guidance. Future studies should leverage social media analysis to gain real-time insight into public health topics of importance in child health and apply targeted interventions.

## Background

The COVID-19 pandemic posed a unique challenge to newborn and maternal health due to quarantine measures resulting in a lack of direct, in-person breastfeeding support from healthcare professionals. There is increasing evidence of the detrimental effect of the pandemic on maternal health. A multinational study, conducted by Cuelemans et al. showed increased anxiety and depression among pregnant and breastfeeding mothers during the COVID-19 pandemic [1]. Similar findings were seen across the globe, such as significantly increased postpartum depression in mothers in the USA [2], increased emotional distress and adverse breastfeeding experiences among mothers in Australia [3], and higher antenatal depression scores among mothers in Turkey [4]. These maternal mental health issues may indirectly affect newborn health by altering nutrition practices such as breastfeeding. A study by Brown and Shenker, during the COVID-19 pandemic, found lower breastfeeding rates among women, with the majority citing lack of face-to-face support as an important cause for breastfeeding cessation [5].

Successful breastfeeding depends on timely education and support for the mothers during the immediate newborn period. In the digital age, women are increasingly turning to online resources and social media sites for this information [6]. With over 300 million monthly users, the micro-blogging platform Twitter became a popular medium for healthcare information dissemination as well as obtaining real-time health data using crowdsourcing methods [7]. Recent studies of social media on breastfeeding show that Twitter plays a key role in breastfeeding promotion through influencer networks involving professionals from the scientific community and the public [8, 9]. Twitter has been used to promote breastfeeding through public awareness campaigns across the world [10, 11].

The role of Twitter in various aspects of health promotion, including health education and healthcare research, was highlighted in a recent systematic review [12]. Many insights can be drawn from the analysis of these micro blogs or “Tweets”. Using twitter analysis, researchers have studied the public sentiment on the COVID-19 pandemic [13] and public perceptions on prevention measures such as social distancing [14], vaccinations [15], and potential treatments [16]. Interestingly, a thematic analysis of pregnancy-related tweets identified anxiety and stress with increased isolation and sleep difficulties in pregnant women [17], which correlated with the findings of studies conducted on mental health in pregnant women across the globe.

The above studies suggest that it might be feasible to assess the effect on newborn health through the social media lens by exploring concepts such as breastfeeding. The significant presence of breastfeeding promotion networks on Twitter presents a unique opportunity to investigate this aspect of newborn health during the COVID-19 pandemic. However, the public sentiment on breastfeeding during the COVID-19 pandemic has not been studied thus far. To address this gap, we used Twitter to study public perceptions and sentiments on breastfeeding during the COVID-19 pandemic.

## Methods

### Data collection and processing

On 19th May 2022, using Twitter’s Application Programming Interface (API) (“Tweepy”. 2022), we accessed Twitter’s COVID-19 stream and collected 40,628 English-language tweets from January 16th, 2020, to May 19th, 2022, related to COVID-19 and breastfeeding. The tweets contained a combination of keywords related to COVID-19 and breastfeeding, such as “newborn feeding,“ “baby feeding,“ “infant feeding,“ and “breastfeeding.“ We also collected 41,599 tweets regarding maternal health using the combination of keywords “maternal health”, “pregnancy”, “COVID” during the same study period. We used Python version 3.9.1 software (Python Software Foundation, Wilmington, DE) [18] for all data processing and analyses. The study did not require Institutional review board approval as we used only publicly available data.

### Tweet characteristics

We used the python library M3-Inference [19], a deep learning model that uses a Twitter user’s profile image, screen name, first and last name, and profile descriptions to predict the type of account and other tweet characteristics.

### Sentiment & emotion analysis

Before performing sentiment analysis, we preprocessed the tweets into their plain text form, which required the removal of hyperlinks, URLs, Twitter handles, “#” symbols, and tweets that were replies. We used the NLTK Library [20] to remove stop words, which are words that provide little semantic meaning to sentiment, such as “their,“ “who,“ and “is.“ We used Python’s SentiStrength Library [21] to identify and classify these preprocessed tweets’ sentiment (positive or negative). SentiStrength is a lexicon-based classifier and a rule-based algorithm to measure sentiment on a scale of − 4 (most negative) to 4 (most positive). We calculated the mean weekly sentiment for all tweets and the average sentiment for each topic.

Before performing an emotion analysis of the tweets, we needed to clean the preprocessed tweets further. We used the Spacy library [22] to clean the text by tokenizing it into individual words. We then transformed the tokens into their root form through natural language processing techniques such as lemmatizing and removing of non-alphanumeric characters. We used the Python library NRCLex [23] to label the primary emotion for each tweet (fear, anger, anticipation, trust, surprise, positive, negative, sadness, disgust, or joy).

### Topic modeling

Using the gensim library in Python [24], we applied the Latent Dirichlet Allocation (LDA) algorithm, an unsupervised machine-learning algorithm, to group tweets using a representative set of words into word clusters. We determined the content of each topic by analyzing these word clusters. We trained the LDA models from 2 to 50 topics to optimize the number of topics in our analysis. We evaluated them based on their topic coherence score, which summarized the semantic similarity among high-scoring (frequent) words within topics. We ultimately chose a 6-topic LDA model that produced the highest score. An author (CUL) without access or insight into the topic modeling labeled the topics using the 30 most frequently used terms, ranked by weight. A subset of authors (JJ, RJM) then evaluated the topic labels to reach a consensus and identified example tweets whose content pertained > 99% to a specific topic.

## Results

### Tweet characteristics

Of 28,216 users, 3,679 had verified accounts (13.0%). Table 1 shows the user and tweet characteristics. The vast majority of tweets came from personal accounts (60.3%). There were 40,628 unique tweets posted by 28,216 (1.4 tweets per user). Twitter for Android was the most used platform (27.8%), followed by the Twitter Web app (27.4%) and Twitter for iPhone (26.0%) (Table 1). The tweets were retweeted (16.1%) more often than liked (14.3%), replied to (5.6%), or quoted (4.4%).

**Table 1:** Characteristics of tweets

**Tweets**	40,628	
**Users**	*n* = 28,216	
**Verified Twitter account**	3,679	
**Followers (Sum [1st – 3rd quartile])**	47,562 [187–4368]	
**Posts to date (Sum[1st – 3rd quartile])**	49,238 [1,933–32,371]	
**Type of account**		
Individual	17,014 (60.3%)	
Organization	11,202 (39.7%)	
**Characteristics**:	# of Tweets	(%)
Has reply	2,284	5.6
Has like	5,817	14.3
Has retweet	6,548	16.1
Has been quoted	1,802	4.4
**Twitter Source**:		
Twitter for Android	11,325	27.8
Twitter Web App	11,132	27.4
Twitter for iPhone	10,591	26.0
Hootsuite Inc.	1,470	3.6
TweetDeck	720	1.8
Other Sources	5390	13.4

*IQR* Interquartile Range

### Emotion and sentiment analysis

**Fig. 1:**
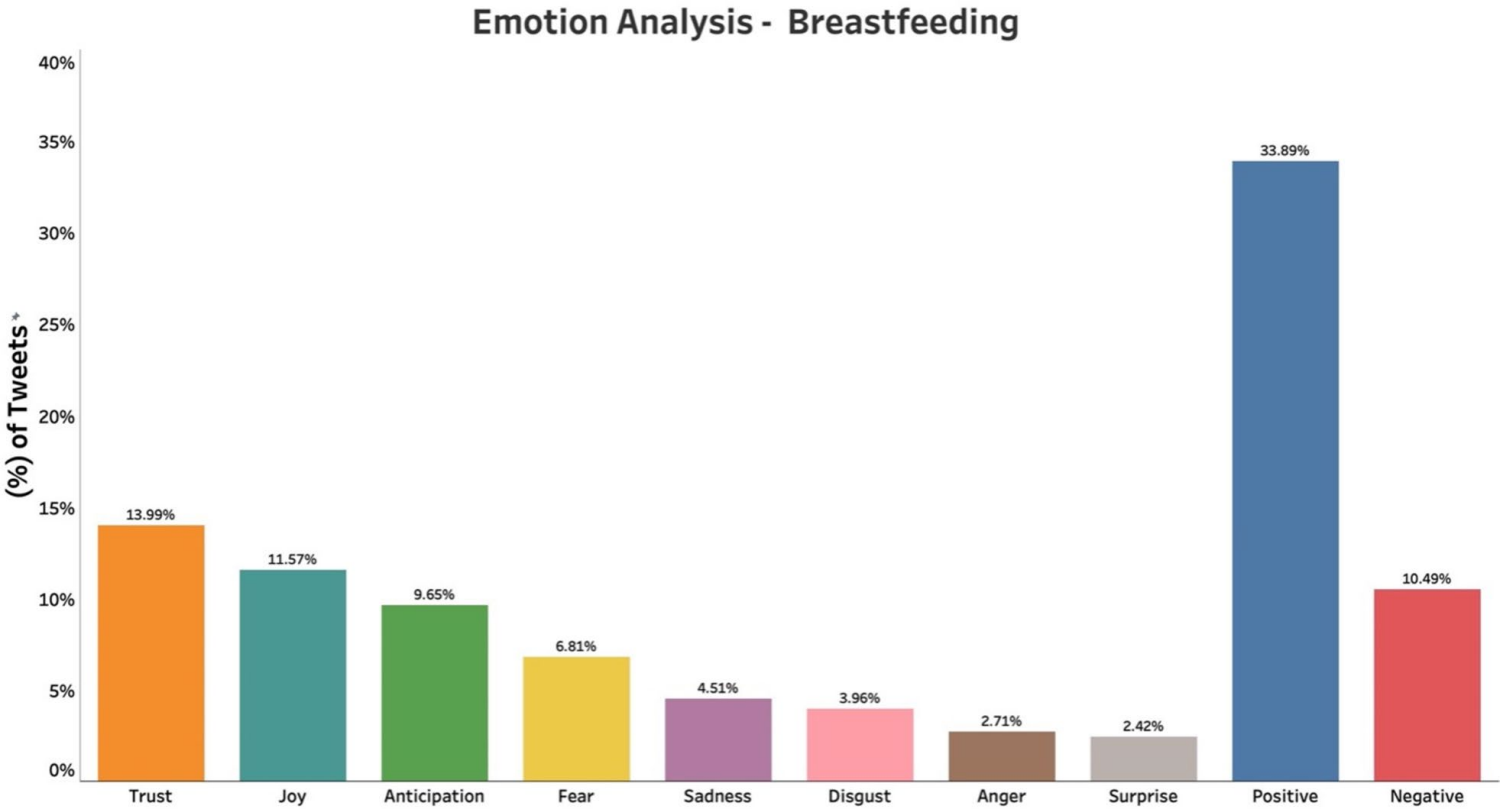
Emotion analysis of tweets related to breastfeeding during the COVID-19 pandemic. Positive emotions, trust, and joy, constituted most of the tweets

**Fig. 2:**
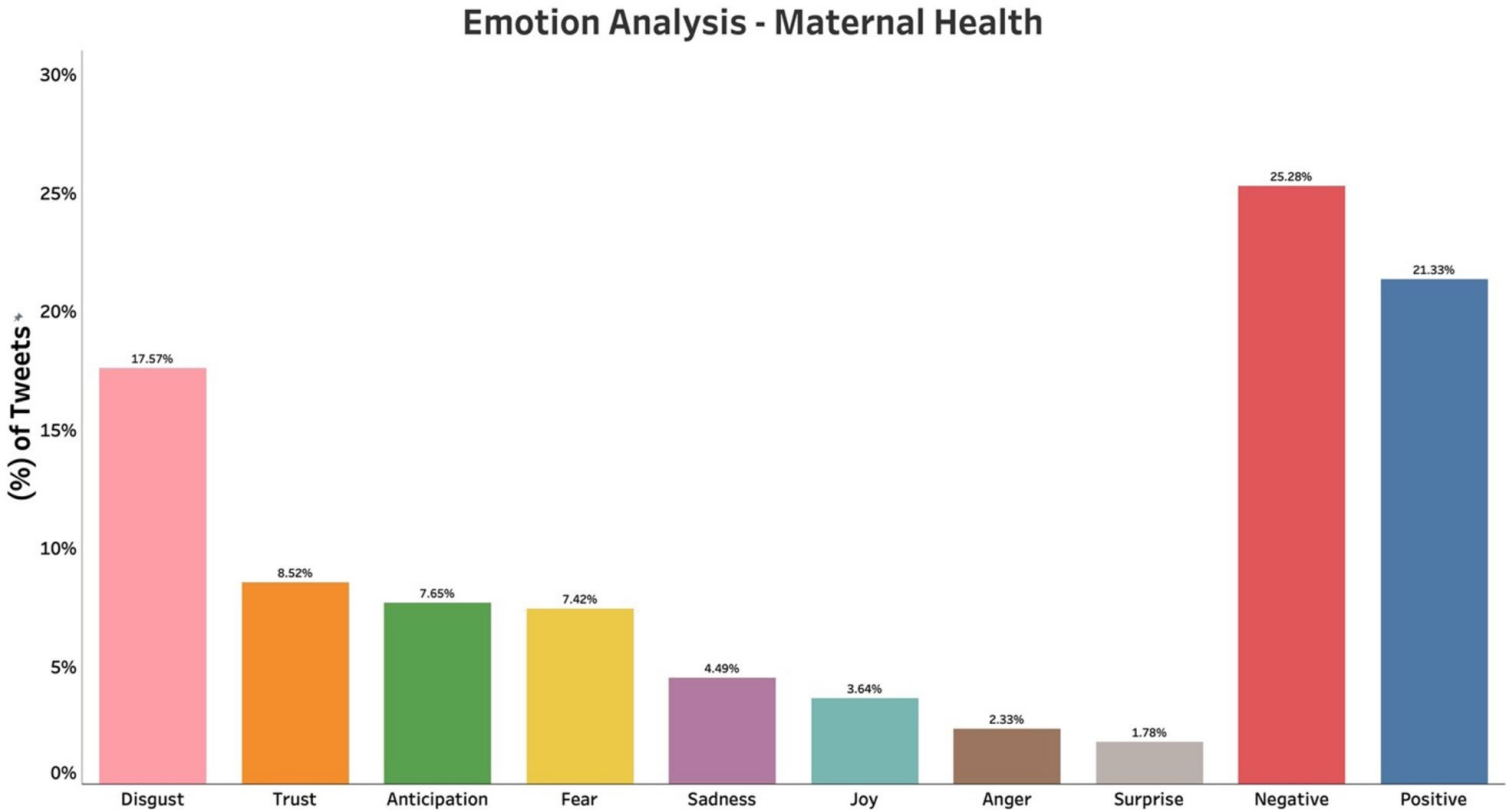
Emotion analysis of tweets related to pregnancy and maternal health during the COVID-19 pandemic. Negative emotions constituted most of the tweets

We noted predominantly “Positive emotions” (Positive, Trust, Joy, Anticipation, Surprise) associated with breastfeeding throughout the study period with 72% of tweets. Negative emotions (Negative, Fear, Sadness, Disgust, Anger) comprised the rest of the tweets (28%) (Fig. 1). Positive emotions are reflected in most tweets (33.8%), followed by Trust (13.9%), Joy (11.5%), and negative (10.4%) emotions. In contrast, the emotion analysis of tweets (n = 41,599) collected during the same period regarding maternal health, and pregnancy during the pandemic showed mostly “Negative emotions” (Negative, Disgust, Fear, Sadness, Anger) comprising 57% of the tweets (Fig. 2).

**Fig. 3:**
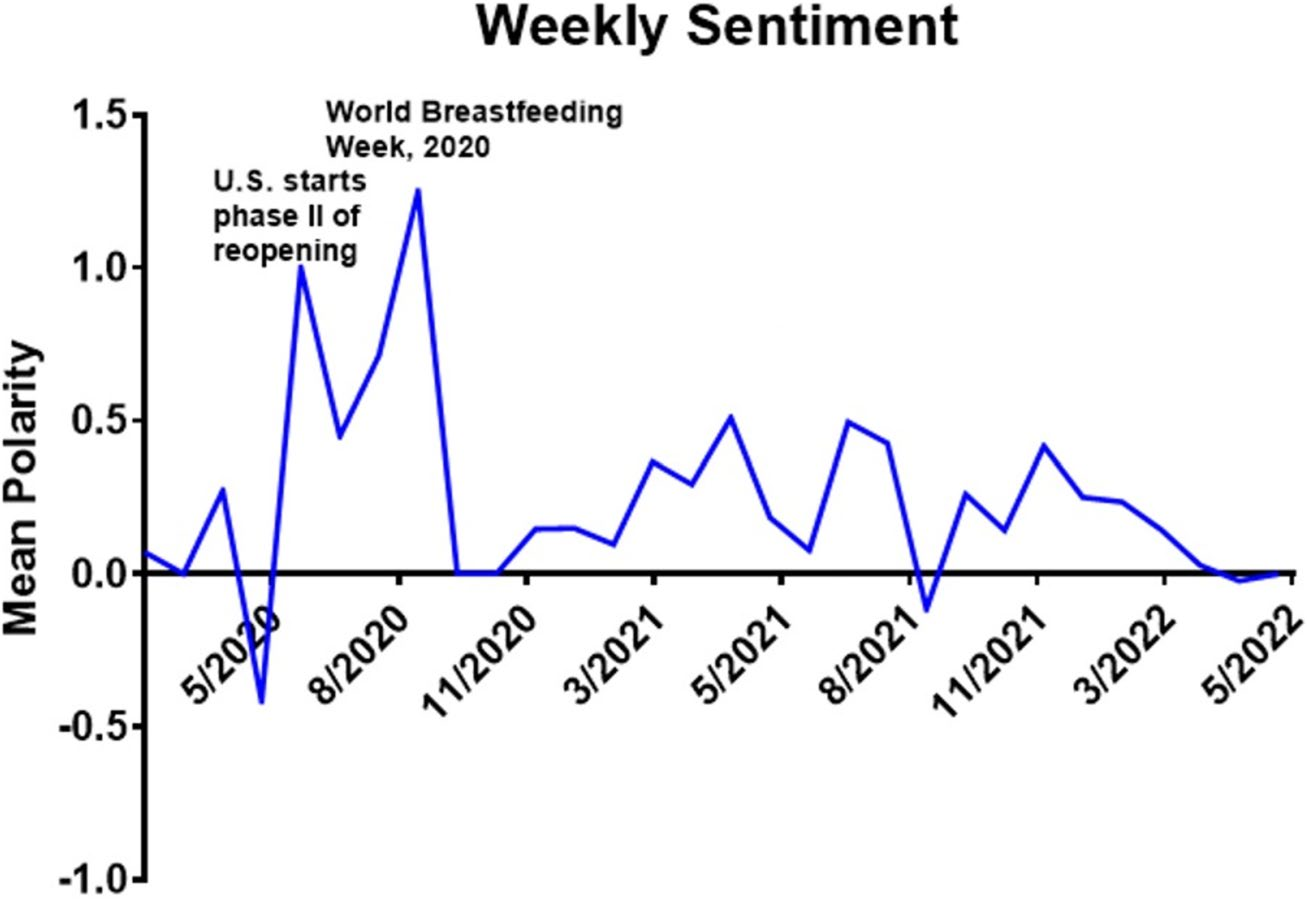
Sentiment analysis of tweets related to breastfeeding during the COVID-19 pandemic. The mean weekly sentiment was 0.25 during the study period. Positive sentiment spikes were seen during the reopening following the lockdown and during the weeks following “World Breastfeeding Week” in 2020

The overall tweet sentiment for breastfeeding was positive, with a mean weekly sentiment of 0.25 through the study period, as noted in Fig. 3. During the week of June 21st, 2020, the United States began phase II of reopening the country, in which the polarity of the tweets became very positive. We found a continued increase in sentiment polarity through August 2020 towards a peak in the week of September 13th, 2020, with the highest mean polarity of 1.25. The first week of August is celebrated as world breastfeeding week every year, which might have correlated with the increase in positive sentiment polarity. On September 12th, 2021, the polarity became slightly negative for the first time since May 2020, which we could not correlate to any real-world events. The sentiment began trending positively in the subsequent weeks. In contrast, the mean tweet sentiment for pregnancy and maternal health-related tweets during this period was negative (-0.196).

### Topic modeling

The LDA model identified six topics expressed in our sample of tweets, which were labeled subjectively based on their respective keywords (Table 2). “Discussion of Benefits of breastfeeding in the context of “World Breast Feeding Week” “was the most popular topic with 8,044 tweets, which contained tweets that discussed the benefits of breastfeeding. All the topics contained a positive sentiment. “Vaccination may protect babies via breastmilk antibodies” was the topic with the most positive sentiment (mean sentiment score of 0.32), and the “Vaccination while pregnant or breastfeeding” topic was the topic with the least positive sentiment (mean sentiment score of 0.26).

**Table 2:** Topic Modeling for Breastfeeding and COVID-19

Topic	Tweets/ Topic n (%)	Topic Sentiment	Keywords	Representative Tweet
Vaccination while breastfeeding is safe	5,891 (14.5)	0.30	covid, need, know, mom, vaccination, like, question, covidvaccine, vaccine, nature, day, pregnancy, risk, vaccinate, care, coronavirus, thank, pregnant, continue_positive_accord_unicef	Privileged and delighted to receive #CovidVaccine. Reassured by its safety while still breastfeeding. Thank you to the Mater vaccination team, working hard at the weekend. #OurMaterTeam @MaterTrauma https://t.co/bk5zAv7Q3k
Vaccination while pregnant or breastfeeding	7,557 (18.6)	0.26	vaccine, pregnant, woman, vaccination, people, get, protect, covidvaccine, pregnancy, covid, safe, vaccinate, learn, information, recommend, find, child, vikilovesfacs_update_explainer_vaccination, today, fertility_pregnancy_include_trial	Pregnant or breastfeeding? Yet to have the #COVIDvaccine? Don’t miss our pop-up vaccination clinic at BRI’s Women’s & Newborn Unit this Friday, 9-5pm. No appointment necessary! If you’re still undecided, midwives & doctors will be on hand to answer any questions, too. #Bradford
Vaccination may protect babies via breastmilk antibodies	5,851 (14.4)	0.32	vaccine, pregnancy, woman, health, covid, need, pfizer, receive, pandemic, people_receive, let, support, right, coronavirus, time, cdcgov_vaxfact_pregnant, question_talk_healthcare_pro, child, patient_mind_environment_breakthrough, list_pfizer_decision_action	Guidance published in relation to the #coronavirus vaccine and pregnancy and breastfeeding @rgumscap @MidwivesRGU @RGUNMandP @ScottishHV @NHSGrampian @FamilyNhs
Discussion of Benefits of breastfeeding in the context of “World Breastfeeding Week”	8,044 (19.8)	0.29	worldbreastfeedingweek_ideal_food_safe, clean_contain_antibody_help, pr, antibody, vaccinate, vaccine, covid, fertility, pass, safety, woman, pregnant_woman, vaccine_pregnancy_menstruation, medical, pandemic, child, find, ontario_pregnant_immunocompromised, bogochisaac_lower_barrier_vaccination, vaccination	It’s always wonderful to read these amazing #news about #humanmilk. Multiple studies show that there are antibodies in a #vaccinated mother’s milk. #neotwitter #neonatal #NICU #MedTwitter #humanmilk #neonatology #PedsICU @nytimes @heathertal https://t.co/rg3bJFil3M
Supporting Breastfeeding during the COVID pandemic and fighting misinformation	6,541 (16.1)	0.23	covid, pandemic, help, young_child_covid, vaccine_develop, alexberenson_vaer_striking_report, child, worldbreastfeedingweek, vaccine, new, work, today, support, day, unicef_guide_safely_pandemic, woman, post, check, provide, year	Breastfeeding is critical to ensuring child #health, especially during the #COVID19 pandemic. Learn more about the counseling materials experts developed to help #healthworkers and caregivers promote #breastfeeding best practices. #WorldHealthDay2021 https://t.co/ZkePm41mI0https://t.co/24fRQcuWt2
Infant feeding support during COVID	6,744 (16.6)	0.29	vaccine, pregnant_woman, safe, support, pandemic, continue, worldbreastfeedingweek, specific_iga_igg_antibody, study_find_robust_secretion, child, covidvaccine, week_maternal_vacci, help, important, read, family, time, say, vaccinate, covid	WIC is providing remote, no-contact services for families while physically distancing during the #COVID19 pandemic. Services include no-cost nutrition education, breastfeeding support, & referrals to healthcare & other community services! Visit https://t.co/HDGWoZXQtP @SMCHealth https://t.co/Htb1m9tdof

## Discussion

We evaluated the perceptions and sentiments regarding breastfeeding during the COVID-19 pandemic on Twitter. We chose to assess “breastfeeding” in the context of COVID-19 as the topic. Breastfeeding is an essential aspect of newborn health and can be affected by maternal mental health issues, which became more prevalent during the COVID-19 pandemic. The topic modeling identified various breastfeeding-associated discussions on Twitter, such as the safety of breastfeeding, vaccinations and breastfeeding, and passive immunity against COVID-19.

### Breastfeeding: positive sentiment and positive emotions

Contrary to other topics we have examined (e.g., “Scamdemic and Plandemic” or “Ivermectin”) [16, 25], we found overall positive sentiment and emotions about breastfeeding during the pandemic. The overall positive sentiment may be partly due to the finding that this topic was frequently tweeted from Twitter accounts belonging to organizations (39.7% of accounts compared to 10.8% for the topic “Scamdemic & Plandemic”), which promote breastfeeding and tend to use positive emotions in conjunction with this topic. While the overall sentiments of tweets were positive throughout the 24 months of the study, we observed occasional dips into negative sentiment. We found positive spikes in sentiment correlated with real world events such as phase 2 reopening after a lockdown in the US and world breastfeeding week. One negative dip in sentiment correlated with the lockdown in the early periods of the pandemic.

We found that the breastfeeding topic, even in conjunction with COVID-19, was associated with positive emotions on Twitter. Positive emotions, trust, joy, and anticipation, contributed to most of the emotions, which was surprising considering the evidence that maternal mental health issues significantly increased during the pandemic. The predominantly negative emotions and sentiments of maternal health and pregnancy tweets in our study reflected the impact of COVID-19 on maternal health. Similar findings were reported by Talbot et al. [17] who analyzed Twitter discussions on pregnant women in the early stages of the pandemic and found that anxiety, depression, and sleep problems were observed due to isolation. A study from the UK revealed two distinct themes of positive and negative pandemic effects on breastfeeding among mothers. Various factors such as delayed return to work, and greater partner support, contributed to the positive effect on breastfeeding. The negative effect on breastfeeding was more reported among Blacks and other ethnic minorities, who cited lack of social and emotional support and lack of face-to-face support from professionals as major causes [5].

### Breastfeeding, COVID-19, and vaccines

“Breastfeeding and the COVID-19 vaccines” was an important topic of discussion on Twitter and included the subtopics “vaccination during breastfeeding,” “breastfeeding after COVID-19 immunization”, and “benefits of breastfeeding in preventing COVID-19 in infants through passive immunity”. We noted an overall positive sentiment about the topics related to vaccines and breastfeeding during COVID-19. Riad et al. [26] studied COVID-19 vaccine hesitancy among pregnant and lactating women and found that 62.2% of the study population rejected the vaccine recommendation from their physician. In the same study, 61.5% of the population quoted vaccine safety for the child as their priority. The challenges of the pandemic, such as quarantine and lack of face-to-face support from medical professionals, along with skepticism about recommendations, might have contributed to increased reliance on social media for vaccine information and guidance. While social media is beneficial for instant information dissemination, the threat of vaccine misinformation on social media to public health is real [27]. Therefore, there is an essential need for the scientific and public health community to engage and disseminate timely evidence-based advice to the public actively.

### Breastfeeding awareness campaigns

In our study, the largest tweet topic (19.8%) was the overall benefit of breastfeeding. Most of these tweets were in the context of “world breastfeeding week,” observed in the first week of August every year. We noticed an increased number of tweets and a positive spike in the sentiment during world breastfeeding week. Similar findings were seen by another group of researchers, who studied real-time Twitter discussions during world breastfeeding week and found significantly increased tweets by many users and influencers, including members of the scientific community and the public [28]. These yearly campaigns provide an essential venue for knowledge and guidance dissemination on social media.

### Limitations

Our study has a few limitations that must be addressed. First, we used existing tools to analyze the sentiments and emotions of tweets that are not specific to healthcare topics, which could have skewed our analysis. Second, since we targeted only tweets in English and cannot determine users’ geographic location, we are limited in making conclusions about specific countries or countries where English is not the predominant language. Third, the user accounts tweeting about this topic had an unusually high percentage of organizational accounts. As a result, tweets are less likely to represent organic discussions and more likely to represent public health promotion.

## Conclusions

The sentiment and public perceptions about breastfeeding during the COVID-19 pandemic were predominantly positive. Twitter was extensively used during the pandemic to discuss various breastfeeding-related issues such as vaccinations, the safety of breastfeeding during COVID-19, and the overall benefits of breastfeeding. Twitter and other social media sites present a unique opportunity for real-time surveillance of public health-related topics. Dissemination of evidence-based guidance focused on those topics has the potential to influence general practices such as breastfeeding or infant feeding. Future studies in maternal and child health should consider social media analysis as an important tool to understand public health topics of interest.

## Data Availability

All the relevant data was presented in the manuscript. Additional Data can be made available upon request from the corresponding author.
